# A Dual-FBG Sensor with Machine Learning for Microstrain–Temperature Decoupling Under Cyanoacrylate Bonding Toward Catheter Applications

**DOI:** 10.3390/mi17060682

**Published:** 2026-05-30

**Authors:** Sung-Ho Yang, Cheng-Kai Yao, Amare Mulatie Dehnaw, Yong-Quan Zhuang, Peng-Chun Peng

**Affiliations:** Department of Electro-Optical Engineering, National Taipei University of Technology, Taipei 10608, Taiwan; t114659003@ntut.edu.tw (S.-H.Y.); t111659004@ntut.org.tw (C.-K.Y.); mulatieamare7@gmail.com (A.M.D.); t114658013@ntut.org.tw (Y.-Q.Z.)

**Keywords:** fiber Bragg grating, dual-FBG sensing structure, machine learning, strain and temperature measurement, cross-sensitivity compensation, catheter system

## Abstract

In cardiovascular interventional procedures, real-time, precise monitoring of minute strain and temperature fluctuations at the catheter tip is essential to improving both the safety and efficacy of these interventions. Fiber Bragg grating (FBG)-based sensors present a promising solution owing to their diminutive size and immunity to electromagnetic interference; however, the inherent cross-sensitivity between strain and temperature remains a significant obstacle. This paper introduces a dual-FBG fiber optic sensing structure that leverages machine learning techniques. The system incorporates two FBGs: one set acts as the primary sensing element, positioned within a simulated catheter and affixed to the substrate under examination with cyanoacrylate adhesive to detect composite strain and temperature signals; the second set is spirally wound around the catheter surface to solely measure temperature, thus effectively isolating temperature interference. Additionally, a machine learning model is employed to learn the nonlinear mapping between the recorded FBG spectra and the actual strain and temperature parameters. Experimental validation was conducted within the physiologically relevant temperature range of 20 °C to 45 °C. The findings indicate that the proposed machine learning model can successfully decouple strain and temperature, achieving high-precision predictions even in situations where the sensing unit exhibits a slight nonlinear response due to adhesive bonding. This study substantiates the feasibility of utilizing machine learning-enhanced dual-FBG structures for multi-parameter sensing in complex environments. The proposed methodology presents a promising avenue for the development of next-generation smart optical fiber sensors intended for application in catheter systems.

## 1. Introduction

Fiber optic sensors have emerged as highly effective measurement tools in both industrial and medical domains owing to their numerous advantages, which include compact size, lightweight construction, high sensitivity, rapid response times, and inherent immunity to electromagnetic interference [1,2]. These distinctive attributes render them an ideal alternative to conventional electronic sensors, particularly in environments where traditional sensors encounter challenges such as electromagnetic noise, complex wiring, or spatial limitations [3,4,5,6].

In the field of civil engineering, fiber optic sensors have been extensively utilized for the structural health monitoring of large-scale infrastructure, including bridges, tunnels, pipelines, and highways [7,8]. For instance, the integration of FBG sensors within bridge structures facilitates real-time monitoring of strain and temperature, thereby enabling early damage detection and promoting long-term operational safety [9,10,11].

In biomedical applications, the miniaturization and biocompatibility of fiber optic sensors have led to their deployment in various innovative areas [1,12]. One significant application is in catheter-based interventional therapy, where FBG sensors are incorporated into guidewires and catheters. This integration offers real-time feedback on shape, force, and temperature during minimally invasive procedures [13,14]. Recent research indicates that FBG-based vascular shape reconstruction achieves high accuracy comparable to computed tomography (CT) imaging in animal models while effectively eliminating radiation exposure [15]. Additionally, FBG force sensors have found extensive use in robot-assisted cardiac interventions, where their distal force-sensing capabilities enhance surgical safety [16].

Despite the inherent cross-sensitivity of FBG sensors to strain and temperature [17], accurately differentiating between these two parameters in practical applications continues to pose a significant challenge. To address this issue, a variety of compensation techniques have been proposed. Among these, the dual FBG configuration is the most prevalent and straightforward. In this configuration, one sensor is isolated from strain influences and utilized solely as a temperature reference. Additionally, alternative fiber architectures and packaging methods have been explored [18,19].

Dual FBG structures have been extensively utilized for the purpose of temperature compensation. However, the majority of current methodologies are predicated on the fundamental assumption that the sensing FBG demonstrates a linear and repeatable response to strain and temperature variations [20,21]. This assumption holds reasonably well when the FBG is either embedded in a well-designed package or bonded with adhesives that have carefully matched thermomechanical properties, such as modified acrylates or specialized epoxies [22,23]. In these optimized systems, conventional linear matrix inversion techniques or polynomial regression methods typically suffice to effectively decouple the influences of strain and temperature with an acceptable degree of accuracy [24,25].

In numerous practical engineering and medical contexts, it is often impossible to guarantee ideal bonding or packaging conditions. Field technicians and assemblers of medical devices frequently prefer cyanoacrylate instant adhesives, commonly referred to as “super glue”, due to their ease of application, rapid curing time, and strong initial adhesion [26,27]. However, this practical choice introduces various complexities that have been largely neglected in the existing literature. Research conducted by Hopf et al. [28] demonstrated that the use of epoxy adhesives can induce birefringence and viscoelastic stress relaxation, which may contribute to significant measurement uncertainties, particularly under conditions of thermal cycling. This challenge is even more pronounced with cyanoacrylate adhesives owing to their distinctive curing mechanisms and thermomechanical properties.

Cyanoacrylate forms a linear thermoplastic polymer upon curing, which exhibits a glass transition temperature in the range of 140–150 °C [29]. While the existing literature has extensively documented the substantial mechanical degradation of this adhesive at elevated temperatures exceeding 80 °C, a systematic investigation into its viscoelastic behavior within the lower temperature range of 20–45 °C—along with the resulting impact on strain transfer efficiency in surface-attached FBG sensors—has yet to be conducted.

Recent studies have explored the application of machine learning in FBG sensors to achieve strain–temperature decoupling. Xin et al. [30] proposed a causal machine learning framework for temperature compensation of dual FBGs; Wei et al. [31] developed a model based on a convolutional neural network-bilayer long short-term memory network (CNN-BiLSTM) for spectral decoupling; and Deng et al. [32] introduced a deep learning algorithm called ADPNet for strain and temperature decoupling. However, none of these studies specifically addressed the nonlinear effects caused by cyanoacrylate (instant adhesive) bonding under thermal cycling conditions, although it is a widely used viscoelastic adhesive. In addition, few studies have considered the practical limitation of placing two FBGs in very close proximity (<1 cm) within a catheter, as the internal space of the catheter is extremely limited. Placing two fiber Bragg gratings (FBGs) in proximity ensures that their local temperature distributions are nearly identical, thereby minimizing errors caused by spatial temperature gradients—a critical yet often overlooked factor in catheter-based applications, where uneven heating or blood flow can lead to local temperature variations.

To the best of our knowledge, apart from the studies above-mentioned, no other research has systematically described the strain–temperature coupled response of cyanoacrylate-bonded FBG sensors under thermal cycling conditions, nor has any machine learning method been proposed to model the resulting nonlinearity while taking into account two closely spaced FBGs within the conduit. Specifically, three critical gaps remain unaddressed in the literature:

- Nonlinear Strain–Temperature Coupling: The viscoelastic properties of cyanoacrylate adhesives lead to variations in strain transfer efficiency, which depend on both temperature and the level of applied strain. This results in a nonlinear relationship between applied strain and the resulting shift in Bragg wavelength.
- Irreproducibility Under Thermal Cycling: Although the sensor may return to its original wavelength after cooling, indicating no permanent damage, the response during heating cycles can differ across successive cycles. This variation arises from stress relaxation and the history-dependent behavior of the adhesive material.
- Absence of Data-Driven Compensation Models: Traditional linear compensation methods prove inadequate under these conditions. However, there has been no specific development of machine learning-based solutions to address the unique nonlinearities introduced by cyanoacrylate fixation.

To address existing gaps in the field, this paper introduces a machine learning-enhanced FBG sensing structure designed to simultaneously measure microstrain and temperature within a simulated catheter system. The primary innovations of this research can be categorized into three key areas:

- Experimental Characterization: A systematic investigation of the nonlinear strain–temperature response of cyanoacrylate-bonded FBG sensors is conducted within a temperature range of 20 °C to 45 °C. This range encompasses physiologically relevant conditions pertinent to catheter-based interventions.
- Physical Decoupling Design: The study proposes the introduction of a spiral-wound temperature-compensating FBG, which is mechanically isolated from strain. This design functions exclusively as a temperature reference, thereby facilitating a clear separation of thermal effects from strain measurements.
- Machine Learning-Based Nonlinear Modeling: A machine learning model is developed to capture the nonlinear mapping from the dual-FBG wavelength inputs to the corresponding strain and temperature values. This approach effectively circumvents the necessity for an explicit physical model to describe the adhesive’s complex behavior.

To the best of our knowledge, this research represents the inaugural application of machine learning techniques to mitigate cyanoacrylate-induced nonlinearities in dual-FBG sensors, specifically within the context of catheter-based medical applications. The proposed methodology not only addresses the limitations associated with traditional linear compensation techniques but also offers a pragmatic solution for situations in which optimal bonding conditions may not be assured.

## 2. Experimental Setup

**Figure 1:**
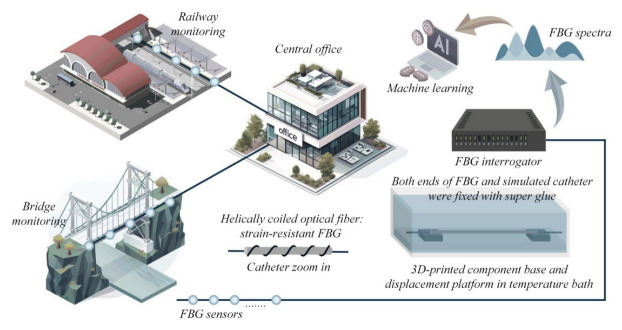
Potential applications for surface-attached sensing and a relative experimental setup for the simultaneous measurement of temperature and strain using dual-FBG.

Figure 1 presents potential application scenarios for the proposed dual-FBG system on the left, alongside the experimental setup designed for simultaneous strain and temperature measurement on the right. In practical implementations, a commercial FBG interrogator transmits interrogation light from a central office to FBG sensors situated at various monitoring locations, such as railways and bridges, to effectively decouple environmental parameters through the analysis of reflected spectra. In the experimental setup, the commercial FBG interrogator (Citpo Technologies Inc., Taipei, Taiwan) directly injects incident light into the corresponding optical fiber to interrogate the FBG at its designated center wavelength. Two FBGs are utilized in this experiment: one is helically wound and affixed to the surface of a 12.3 cm length hollow catheter that has an approximate diameter of 0.7 cm, while the other is extended through the hollow catheter and securely adhered along with the catheter to the displacement platform using the cyanoacrylate adhesive. A 3 D-printed base is positioned beneath the displacement platform to provide stabilization, facilitating FBG strain measurement experiments across various temperatures within a temperature-controlled water bath. The FBG that is spirally wound around the surface of the simulated catheter exhibits temperature sensitivity exclusively and remains unaffected by strain. The initial center wavelength of this FBG is recorded at 1536.04 nm. Conversely, the FBG situated within the interior of the catheter is responsive to both temperature and strain, with its initial center wavelength noted at 1534.02 nm. Both FBGs employed in this study are conventional in design, exhibiting approximately 0.011 nm of wavelength shift per 1 °C variation in temperature, and approximately 1 pm shift per 1 με change in strain. The two FBGs are arranged in series; the optical power at the peak of their reflection spectra varies with the different fusion splices, but this will not affect the outcome of the experiment. On the other hand, the collected spectral data undergo preprocessing before analysis with machine learning models, thereby facilitating the decoupling of the FBG wavelength. The models proposed in this context were developed using Python version 3.8.20 and the Keras library with the TensorFlow backend 2.13.1. All experimental procedures were performed on a computing platform endowed with an 11th Generation Intel^®^ Core™ i7-11700 CPU operating at 2.50 GHz, accompanied by 32 GB of RAM and Intel^®^ UHD Graphics 750.

## 3. Experimental Results

**Figure 2:**
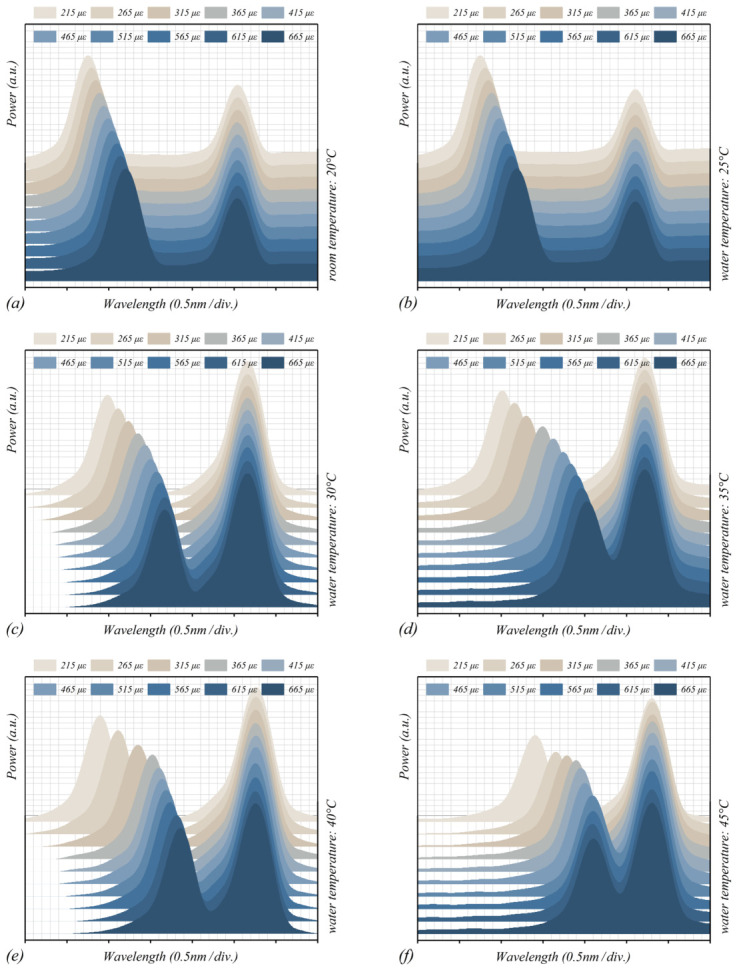
(**a**–**f**) Raw reflectance spectra of the dual-FBG sensor under different strain (215–665 µε) and temperature conditions for (**a**) 20 °C, (**b**) 25 °C, (**c**) 30 °C, (**d**) 35 °C, (**e**) 40 °C, and (**f**) 45 °C. These spectra constitute the input features for training the machine learning model. Although the information in the figures appears dense due to the overlap of multiple spectral curves, the purpose is to qualitatively illustrate the evolution of the spectra under different experimental conditions. Figure 3 and Figure 4 provide a quantitative analysis of the center wavelength shift and strain–temperature decoupling.

Figure 2 illustrates the spectral evolution observed in a dual-FBG sensor that simultaneously measures strain and temperature. The temperatures associated with Figure 2a–f are 20 °C, 25 °C, 30 °C, 35 °C, 40 °C, and 45 °C, respectively. The corresponding strain-free FBG wavelengths recorded at these temperatures are 1536.04 nm, 1536.1 nm, 1536.16 nm, 1536.22 nm, 1536.26 nm, and 1536.31 nm. The helically wound FBG is loosely wound along the helical segment on the conduit surface without the use of adhesives; only the ends are secured with cyanoacrylate to prevent detachment. This loose winding decouples the FBG from the axial strain of the conduit, as the helical path can accommodate length variations without stretching the fiber. Experimental verification shows that no measurable wavelength shift was detected under strains up to 665 µε, while the temperature sensitivity remained at 0.011 nm/°C. Conversely, for another FBG subjected to both strain and temperature variations, the applied strain ranged from 215 με to 665 με, with increments of 50 με at each step. Analysis of the FBG spectra indicates that the drift trajectories exhibit a clear linearity at temperatures of 20 °C and 25 °C; however, nonlinear behavior begins to manifest when temperatures exceed 30 °C. It can be deduced that this phenomenon is not attributable to the loosening of fiber connections at both extremities of the FBG. This conclusion is supported by the observation that upon completing the experiment at 45 °C and subsequently returning the system to room temperature for further evaluation, the spectral change exhibited a linear trend that closely resembled the results presented in Figure 2a. It is worth emphasizing that the machine learning model in this study was trained directly on the raw reflectance spectrum (as shown in Figure 2), rather than on pre-extracted center wavelength values. Unlike traditional wavelength-based methods, which discard spectral shape information such as bandwidth, sidelobes, or asymmetry, the raw spectrum fully preserves the optical response of the FBG sensor. This is particularly important when the sensor exhibits nonlinear or irreversible behavior due to the viscoelasticity caused by the adhesive, in which case, changes in the spectral shape may carry additional information about the adhesive state or strain–temperature coupling. Therefore, Figure 2 comprehensively illustrates the input data used for model training, including subtle variations under different strain and temperature conditions.

**Figure 3:**
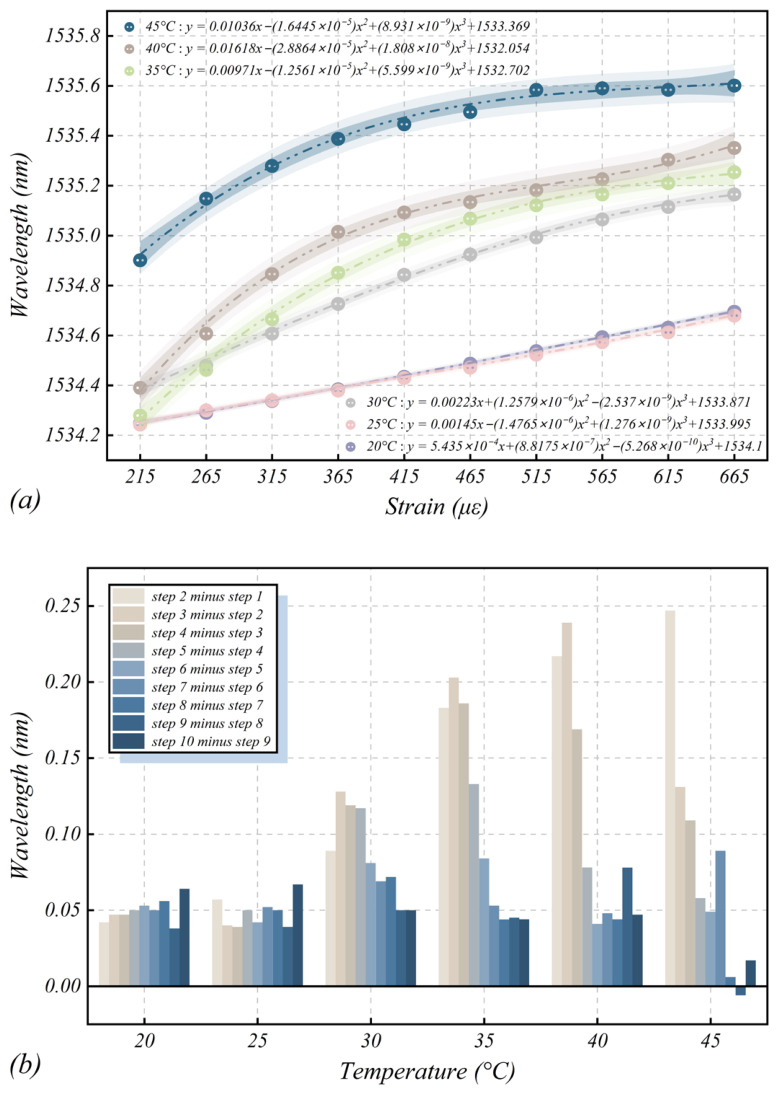
(**a**) The center wavelengths of the FBG were measured across a range of wavelengths from 215 με to 665 με under varying temperature conditions. (**b**) The variations in center wavelengths of the FBG correspond to incremental strain steps at different temperatures.

For a more detailed examination, the center wavelengths of the strained FBG corresponding to the data presented in Figure 2 are illustrated in Figure 3. Figure 3a displays the FBG center wavelengths across strain levels of 215 με to 665 με at the specified temperatures of 20 °C, 25 °C, 30 °C, 35 °C, 40 °C, and 45 °C. In contrast, Figure 3b primarily highlights the variations in the FBG center wavelengths between consecutive strain increments. In the strain measurements conducted at temperatures of 20 °C and 25 °C, the initial wavelength was standardized at 1534.25 nm. As a result, the measured strain outcomes at both temperatures were found to be comparable. Additionally, a shift of approximately 0.05 nm (equivalent to 50 με) was recorded per incremental step, which aligns with the previously established shift of approximately 1 picometer per 1 με change in strain. This finding suggests that the characterization of the cyanoacrylate adhesive at 20 °C and 25 °C does not influence the strain response of the FBG. For the strain measurements conducted at temperatures of 30 °C, 35 °C, 40 °C, and 45 °C, the FBG sensor was initially positioned at room temperature, with its baseline wavelength calibrated to 1534.25 nm prior to immersion in a temperature-controlled water bath. It is evident that the center wavelength of the FBG, while placed within the water bath, does not entirely align with the anticipated wavelength variations attributable to temperature changes. Additionally, during the initial phases of strain application, there is a pronounced wavelength drift, which tends to stabilize in later stages. This phenomenon can be attributed to the immediate thermal expansion of the cyanoacrylate adhesive upon immersion in the water bath, resulting in the optical fiber being subjected to increased tension and a temporary enhancement in strain sensitivity. As the experiment progresses, the wavelength drift gradually diminishes due to stress relaxation of the cyanoacrylate adhesive, likely influenced by viscoelastic slippage and the rearrangement of the cyanoacrylate molecular chains, a trend that is particularly notable at 45 °C. This is because the thermal expansion coefficient of cyanoacrylate, which ranges from approximately 80 to 100 ppm/°C, significantly surpasses that of optical fiber, measured at about 0.55 ppm/°C. Furthermore, it is plausible that localized depolymerization (or unzipping) of the cyanoacrylate occurs upon heating, leading to alterations in the molecular weight distribution, which subsequently impacts the mechanical properties of the adhesive layer and the overall efficacy of strain transmission [29]. Although these microscopic alterations at the polymer level do not induce macroscopic delamination—evidenced by the FBG wavelength returned to normal at room temperature following the experiment—they are sufficient to impede the reproducibility of the FBG sensor response after multiple thermal cycles, as observed subsequent to the second experimental trial. Furthermore, the 3D-printed base of the fixed-displacement platform is composed of polylactic acid (PLA), which does not exhibit thermal deformation that may affect strain measurements until it reaches 55 °C [33,34]. Incidentally, at 45 °C, the cyanoacrylate adhesive layer exhibits significant creep behavior; under equivalent strain conditions, the FBG shifts slightly toward longer wavelengths by 0.03–0.1 nm after 1 min. This creep behavior is indicative of viscoelastic polymers, such as cured cyanoacrylate, wherein molecular chain rearrangement results in delayed deformation under sustained loading. The creep experiment was repeated under the same conditions. After one minute, the wavelength shift varied between cycles (ranging from 0.03 to 0.1 nm) without any consistent pattern. However, the wavelength returned to its baseline value when the sensor was cooled to 25 °C (as shown in Figure 2a). This outcome confirms the non-reproducibility of the cyanoacrylate-bonded FBG response across cycles. Unfortunately, the absence of reproducible patterns hinders the ability to quantitatively model the multi-cycle process in this study.

In this study, extreme gradient boosting (XGBoost) was employed as the principal machine learning model for predicting FBG wavelength responses. The experimental dataset consisted of 180 FBG spectra, collected from six temperature conditions of 20 °C, 25 °C, 30 °C, 35 °C, 40 °C, and 45 °C. For each temperature condition, strain was applied from 215 με to 665 με with 50 με increments, corresponding to 10 strain levels. Each temperature–strain condition was measured three times, resulting in 180 spectra in total. The dataset was divided into training and validation subsets using an 80/20 ratio, corresponding to 144 training spectra and 36 validation spectra. To maintain sample balance, the split was stratified by temperature. In addition, model complexity was controlled using limited tree depth, subsampling, column sampling, and L1/L2 regularization to reduce the possibility of overfitting. The selection of XGBoost was based on its effective management of structured data, its built-in regularization mechanisms, and its exceptional capability to model nonlinear relationships [35,36,37]. The hyperparameters of the model were systematically optimized, resulting in the following settings: the number of decision trees (n_estimators) was established at 1200, the learning rate was set to 0.015, and the maximum tree depth was limited to 4 in order to control model complexity and mitigate the risk of overfitting. Moreover, the minimum child weight (min child weight) was set to 2, while both the subsample and colsample_by_tree parameters were configured to 0.90 to enhance the model’s generalization ability. The regularization parameters L1 and L2 were assigned values of 0.05 and 1.2, respectively, to further restrict model complexity. In terms of feature engineering, the original spectral data were condensed into 64 principal components through principal component analysis, preserving the majority of the variance. Additionally, to capture the nonlinear interactions between strain and temperature, three supplementary engineered features were included: the product of strain and temperature (strain × temperature), the square of strain (strain^2^), and the square of temperature (temperature^2^). As a result, the final input dimension consisted of 69 features, combining the PCA components with the meta-features of strain and temperature. Furthermore, the XGBoost model was used as a nonlinear wavelength–response calibration model. The training dataset included spectral intensity features, strain, temperature, and the corresponding measured FBG wavelength. After preprocessing, the spectral data were reduced using PCA and combined with strain–temperature calibration features to predict the FBG wavelength response. Therefore, the present model is intended to characterize the nonlinear relationship between the applied calibration conditions and the measured FBG wavelength, rather than to serve as a direct inverse sensing model. Future work will extend this framework toward an inverse sensing model that uses only optical spectral signals to estimate unknown strain and temperature.

**Figure 4:**
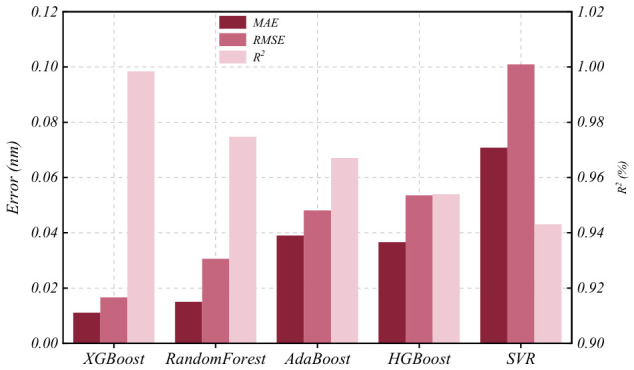
Comparison of XGBoost, random forest, AdaBoost, HGBoost, and SVR with regard to MAE, RMSE, and R^2^ metrics.

**Figure 5:**
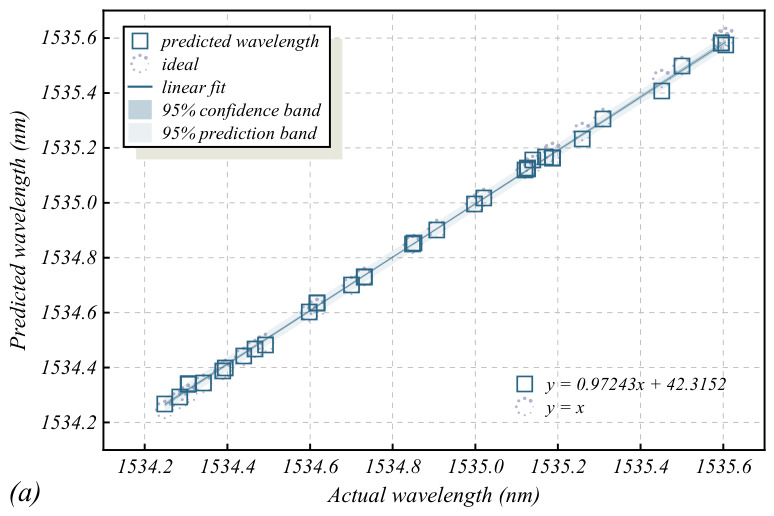

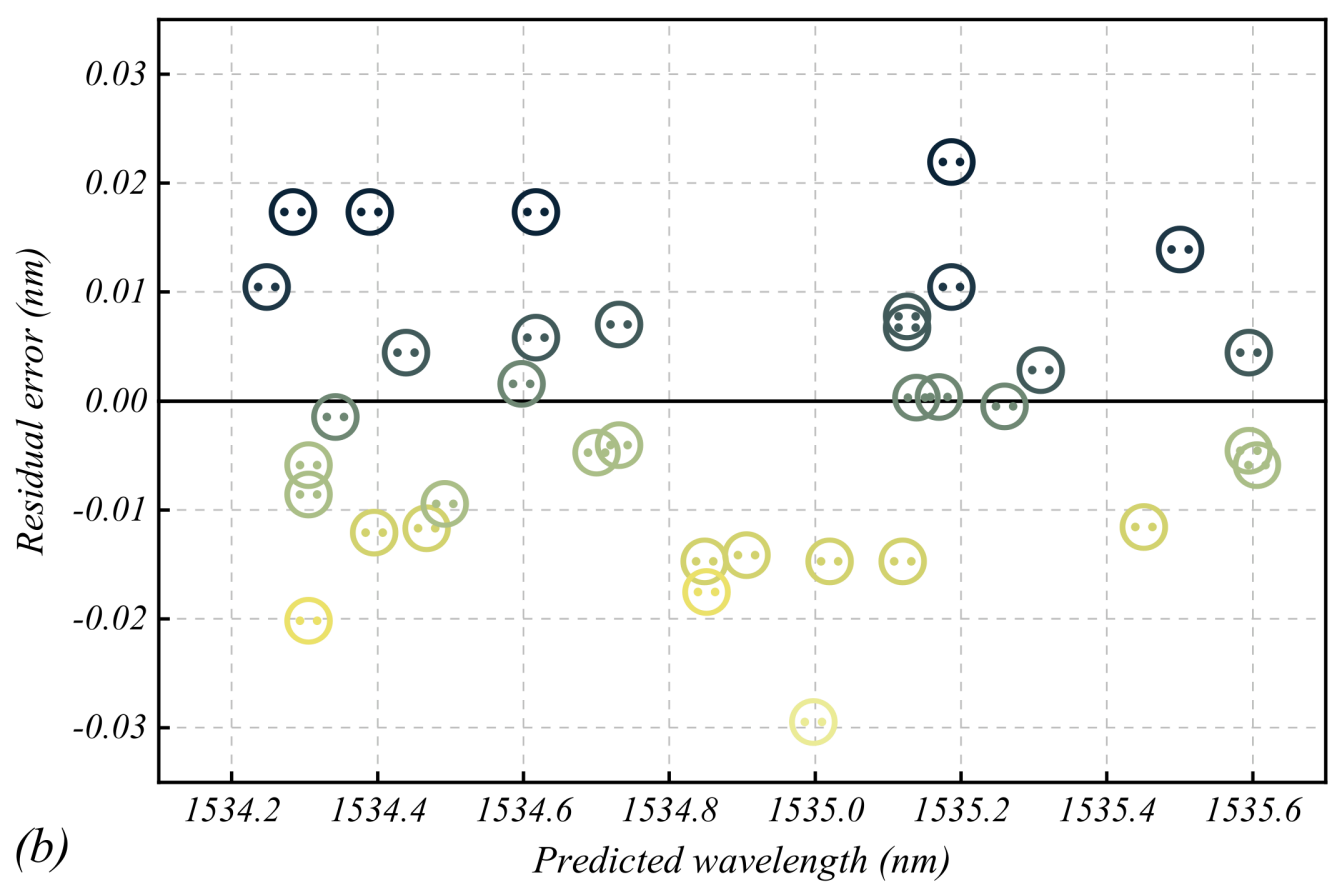
(**a**) Comparison of predicted and experimental wavelength values using the XGBoost model. (**b**) Residual analysis of the XGBoost model includes the prediction error (actual − predicted).

The model utilized root mean squared error (RMSE) as the loss function, with mean absolute error (MAE) and coefficient of determination (R-squared, R^2^) as evaluation metrics. To validate the effectiveness of XGBoost, a comparison was undertaken with random forest, adaptive boosting (AdaBoost), histogram gradient boosting (HGBoost), and support vector regression (SVR). XGBoost demonstrated superior prediction accuracy with an R^2^ of 0.998, a MAE of 0.0111, and an RMSE of 0.01666 across all evaluation metrics, outperforming the other four models, as illustrated in Figure 4. This ranking of performance aligns with established observations within the literature pertaining to structured data [38], further affirming the efficacy of XGBoost in the FBG wavelength prediction task. Figure 5 further provides a comprehensive illustration of the XGBoost model’s prediction results concerning FBG wavelength values. Specifically, Figure 5a compares the predicted wavelengths with the actual wavelengths (under ideal prediction conditions), while Figure 5b shows the residuals between the actual and predicted wavelengths (which align with the MAE presented in Figure 4).

This study validates the predictive capabilities of machine learning models concerning the nonlinear response of cyanoacrylate-fixed FBGs within a singular thermal cycle. However, considering the constraints of the experimental conditions (limited to a single complete thermal cycle) and the viscoelastic properties inherent to cyanoacrylate, it is essential to delineate the scope, limitations, and future directions of this research.

Initially, this study should be regarded as a demonstration of feasibility under specified experimental circumstances (a single thermal cycle). Results indicate that machine learning models, such as XGBoost, can effectively capture the nonlinear mapping relationship between strain and temperature concerning the FBG wavelength, achieving high prediction accuracy in both the training and test datasets. This outcome illustrates the continuing applicability of data-driven methods, even when sensor responses deviate from linearity. The contribution of this work lies in demonstrating the potential of machine learning as a viable alternative to traditional linear calibration methods, particularly in contexts where sensor behavior is complex and challenging to define precisely through physical models.

Secondly, this study observed an intriguing phenomenon that has been rarely documented systematically in the existing FBG literature: FBGs that are surface-mounted using cyanoacrylate exhibit non-repeatability during multiple thermal cycles. Notably, the sensor wavelength reverts to normal after each cycle when tested in air, indicating the absence of permanent damage. This phenomenon may be attributed to the linear thermoplastic polymer structure of polycyanoacrylate and its viscoelastic behavior under thermal conditions, including molecular chain motion, stress relaxation, and potential local unzipping [29]. To the best of our knowledge, this represents the first explicit documentation of such a phenomenon within the FBG sensing literature, establishing a connection to the polymeric properties of the adhesive. The significance of this finding lies in highlighting the fundamental limitations associated with the use of ordinary superglue for FBG fixation, thereby providing a vital caution for engineering practices: in applications that necessitate long-term stability or repeated thermal cycles, it is advisable to utilize adhesives with cross-linked structures or enhanced thermal stability, such as modified acrylics or epoxy resins.

Thirdly, to demonstrate the generalizability of the machine learning framework proposed in this study, supplementary validation was performed using physics-based synthetic data, or a complete presentation of the model architecture and hyperparameter settings was provided for other researchers to replicate and adapt to alternative datasets. The results derived from the synthetic data affirm that the proposed machine learning architecture can effectively learn the nonlinear strain–temperature mapping, even when exposed to varying data distributions. The contribution of this aspect elevates the value of this study from mere “specific experimental results” to the realm of “methodological contributions”, indicating that the machine learning architecture detailed in this work can be applied beyond the specific adhesive type or temperature range utilized in this experiment, making it relevant to various FBG sensing scenarios characterized by nonlinear coupling.

Before clinical translation, several practical challenges must be addressed, including sterilization (e.g., using ethylene oxide or hydrogen peroxide plasma, as high-pressure steam sterilization may cause the adhesive to degrade), biocompatibility (compliance with ISO 10993 standards, which may require the use of a poly(p-xylene-C) coating), dynamic blood flow conditions (which may require real-time filtration or adaptive machine learning models), and miniaturized designs for small-bore catheters. Addressing these challenges is critical for future translational research. Furthermore, assuming a typical catheter stiffness of approximately 500 N/m [39], the strain range of 215–665 µε corresponds to a catheter tip contact force range of approximately 0.1–0.3 N. This range is clinically significant in cardiac ablation procedures: excessive force (>0.4 N) may lead to tissue perforation, while insufficient force (<0.05 N) may result in inadequate lesion formation. Therefore, the selected strain range represents a physiologically significant load condition in catheter-based interventional therapy. However, this experiment was conducted under static, isothermal conditions without considering pulsatile blood flow or cardiac motion; dynamic validation is left for future research.

## 4. Conclusions

This study proposes and experimentally validates a machine learning-enhanced dual-FBG fiber optic sensing structure that is designed to concurrently measure microstrain and temperature within a physiologically relevant temperature range of 20–45 °C for catheter-related interventional therapy. The experimental results indicate that despite the main FBG being bonded with cyanoacrylate adhesive—a commonly used viscoelastic material that introduces nonlinearity into the strain–temperature response—the proposed XGBoost model, trained utilizing PCA-reduced spectral features and an engineered strain–temperature interaction term, achieved a coefficient of determination exceeding 0.998. This performance significantly surpassed that of the random forest, AdaBoost, HGBoost, and SVR models. The helical-wound temperature-compensated FBG effectively isolates thermal effects, thereby enabling the machine learning model to facilitate high-precision strain–temperature decoupling, even in the presence of nonlinear behavior induced by the adhesive. These findings underscore the feasibility of integrating dual-FBG configurations with machine learning for multi-parameter sensing in complex and temperature-variable environments, establishing a practical pathway for the advancement of next-generation intelligent fiber optic sensors for catheter systems.

## Data Availability

The data presented in this study are available on request from the corresponding author.
